# Evaluating the use of statistical and machine learning methods for estimating breed composition of purebred and crossbred animals in thirteen cattle breeds using genomic information

**DOI:** 10.3389/fgene.2023.1120312

**Published:** 2023-05-15

**Authors:** C. A. Ryan, D. P. Berry, A. O’Brien, T. Pabiou, D. C. Purfield

**Affiliations:** ^1^ Teagasc, Co. Cork, Ireland; ^2^ Munster Technological University, Cork, Ireland; ^3^ Irish Cattle Breeding Federation, Cork, Ireland

**Keywords:** genomic breed composition, cattle, crossbred, population assignment, low-density panels, best linear unbiased prediction, Admixture, genetic diversity

## Abstract

**Introduction:** The ability to accurately predict breed composition using genomic information has many potential uses including increasing the accuracy of genetic evaluations, optimising mating plans and as a parameter for genotype quality control. The objective of the present study was to use a database of genotyped purebred and crossbred cattle to compare breed composition predictions using a freely available software, Admixture, with those from a single nucleotide polymorphism Best Linear Unbiased Prediction (SNP-BLUP) approach; a supplementary objective was to determine the accuracy and general robustness of low-density genotype panels for predicting breed composition.

**Methods:** All animals had genotype information on 49,213 autosomal single nucleotide polymorphism (SNPs). Thirteen breeds were included in the analysis and 500 purebred animals per breed were used to establish the breed training populations. Accuracy of breed composition prediction was determined using a separate validation population of 3,146 verified purebred and 4,330 two and three-way crossbred cattle.

**Results:** When all 49,213 autosomal SNPs were used for breed prediction, a minimal absolute mean difference of 0.04 between Admixture vs. SNP-BLUP breed predictions was evident. For crossbreds, the average absolute difference in breed prediction estimates generated using SNP-BLUP and Admixture was 0.068 with a root mean square error of 0.08. Breed predictions from low-density SNP panels were generated using both SNP-BLUP and Admixture and compared to breed prediction estimates using all 49,213 SNPs (representing the gold standard). Breed composition estimates of crossbreds required more SNPs than predicting the breed composition of purebreds. SNP-BLUP required ≥3,000 SNPs to predict crossbred breed composition, but only 2,000 SNPs were required to predict purebred breed status. The absolute mean (standard deviation) difference across all panels <2,000 SNPs was 0.091 (0.054) and 0.315 (0.316) when predicting the breed composition of all animals using Admixture and SNP-BLUP, respectively compared to the gold standard prediction.

**Discussion:** Nevertheless, a negligible absolute mean (standard deviation) difference of 0.009 (0.123) in breed prediction existed between SNP-BLUP and Admixture once ≥3,000 SNPs were considered, indicating that the prediction of breed composition could be readily integrated into SNP-BLUP pipelines used for genomic evaluations thereby avoiding the necessity for a stand-alone software.

## Introduction

While genomic information in livestock breeding and management has predominately been used for parentage verification and discovery as well as genomic evaluations, it also has other potential applications such as the prediction of breed composition (Kuehn et al., 2011; Mcclure et al., 2017). In the absence of genomic information, the breed proportion of an animal is assumed to be simply the average breed composition of both parents (Sölkner et al., 2010). However, breed composition of the offspring from a crossbred parent may deviate from expectation owing to parental recombination of chromosomes during gametogenesis. Genomic information should be more precise in predicting the breed composition of animals due to its capacity to determine the parental contribution (Strucken et al., 2017; Kumar et al., 2021) and therefore can help correct pedigree errors and estimate kinships when ancestry data are missing.

The ability to accurately predict the breed composition of an animal using genomic information has many potential uses. Firstly, genomic information can be used to verify that an animal is a purebred, thus preserving the integrity of the herd book. This may be of particular benefit for rare breeds where limited purebred breeding individuals exist or where no pedigree information is recorded and, therefore, it can help correct pedigree errors and estimate kinships when ancestry data are missing. Secondly, prediction of breed composition could assist in delivering consumer confidence in the authenticity of products from certain breeds which may command a higher market price (Judge et al., 2017; O’Brien et al., 2020). Furthermore, where service providers genotype animals from multiple breeds, comparing breed composition estimates from genotype data against the expected breed composition for a given genotyping plate could curtail pedigree errors and act as a quality control measure by identifying mislabelled genotypes prior to their inclusion in downstream analyses (Kumar et al., 2021). While sex is a routine quality control step in the genotyping process, using breed composition prediction as an additional quality control measure may be particularly useful if a plate of exclusively male or female genotypes is mis-oriented. Additionally, the accurate determination of an animal’s breed composition may improve the robustness of genetic evaluations where breed composition is frequently employed as an adjustment factor (Thomasen et al., 2013; McHugh et al., 2017), to correct for the differences in allele frequency and the relationship between SNPs and quantitative trait loci across breeds. Indeed, due to the mosaic nature of a crossbred animal’s genome, Sevillano et al. (2017) confirmed that accounting for breed-specific SNP effects in admixed genomic evaluations outperformed genomic prediction models where the SNP effects were assumed to be the same across breeds. This suggests that the accurate determination of breed composition can enhance genomic predictions.

The SNP-BLUP method routinely used in genomic evaluations can also be used to predict breed composition, as proposed in sheep by O’Brien et al. (2020). By in large, the SNP-BLUP approach uses an infinitesimal model which assumes that the trait of interest is controlled by large number of SNPs, each of very small effect, fitted as random effects with a common variance structure. As SNP-BLUP is often used in genomic evaluations, the ability to exploit existing pipelines for predicting breed composition could be advantageous for quality control and be more computationally efficient than using a stand-alone software for breed prediction. Therefore, the objective of the present study was to use a large database of genotyped purebred and crossbred cattle to compare breed composition predictions using a freely available software, Admixture (Alexander et al., 2009), with those from SNP-BLUP. While statistical metrics and methods such as F_st_ and PCA have been used to select informative SNPs to discriminate between cattle breeds (Wilkinson et al., 2011; Hulsegge et al., 2013), we wanted to determine the effectiveness of these methods in particular for identifying informative SNPs for predicting crossbred breed composition. Moreover, we aimed to compare the performance of these methods against other SNP selection approaches, including machine learning algorithms. Therefore, an additional objective was to determine the accuracy and general robustness of low-density genotype panels for predicting breed composition which was achieved by varying 1) the SNP density, and 2) the SNP selection strategy for alternative custom-derived low-density panels.

## Materials and methods

### Genotypic data

A total of 52,655 SNP were available from 703,078 dairy and beef cattle generated using a custom Illumina beadchip (IDBV3) which was developed to primarily increase the accuracy of genomic predictions whilest generating genotype information for mutations of interest (Mullen et al., 2013). All animals had a call rate ≥90%. Only autosomal SNPs, SNPs with a known chromosome and position on the ARS UCD 1.2 genome build, and those with a call rate ≥ 90% were retained. SNPs were not filtered based on minor allele frequency to ensure that informative SNPs for distinguishing breeds with lower numbers were not omitted. Following all edits, 49,213 SNPs from 703,078 animals remained. Sporadically missing genotypes were imputed using FImpute V2.2 which uses an overlapping sliding window approach to efficiently exploit both family and population based information (Sargolzaei et al., 2014).

### Establishment of purebred populations

Expected breed composition was available on all animals based on their recorded ancestry; 98,883 genotyped animals from 13 breeds were expected (based on ancestry) to be purebred. Breeds included were Angus, Aubrac, Blonde d'Aquitaine, Belgian Blue, Charolais, Friesian, Hereford, Holstein, Limousin, Parthenaise, Saler, Shorthorn, and Simmental. Using the available genotypes, a principal component analysis (PCA) based on a genomic relationship matrix was calculated using the approaches described by Yang et al. (2011) in the GCTA software package (Yang et al., 2011) to ensure animals were recorded correctly as being purebred. The 49,213 SNPs were pruned prior to PCA analysis by excluding one SNP from a pair of SNPs in strong linkage disequilibrium (pairwise squared correlation $r^{2}$ > 0.5) in a chromosomal window size of 50 SNPs, sliding the window 10 SNPs at a time as suggested by Dutheil (2020) to ensure that the resulting components were representative of the true underlying structure in the data and to reduce the risk of over-representation of certain regions of the genome; a total of 22,606 SNPs remained. Animals that deviated from their respective breed cluster in the PCA plot based on principal components 1, 2, and 3 were deemed to be incorrectly recorded as being purebred resulting in 11,210 animals being discarded.

Admixture V1.3 (Alexander et al., 2009) was also used to verify each animal’s breed composition using the 22,606 pruned SNPs dataset as suggested by Alexander et al. (2009). The pruned dataset was used solely to verify purebred status but the full SNP dataset was used for breed prediction analyses and SNP selection. An unsupervised analysis was initially performed to determine the most appropriate number of breed clusters (*K*) from 11 to 14. *K* = 13 was the chosen number of breed clusters as it had the lowest cross-validation error; each of the 13 breeds separated into a distinct cluster. Individuals with a subsequent ancestry assignment of ≥ 90% attributed to one breed were retained as purebred-verified animals. The 44,802 purebred-verified animals were subsequently available to be stratified into three separate populations for analysis; 1) a training population, 2) a purebred validation population, and 3) a third purebred population which we will refer to as the SNP selection population; each population served a unique purpose described later. Given that some breeds had more purebred animals than other breeds, not all 44,802 purebred animals were used in the analysis; this was to ensure the number of animals selected per breed was relatively similar in order to minimise bias. A summary of the number of animals per breed within each of the three purebred populations is shown in Table 1.

**TABLE 1:** Number of animals per breed within the purebred training, validation and SNP selection populations.

Cattle breed	Training	Purebred validation	SNP selection
Angus	500	250	1000
Aubrac	500	250	1000
Blonde d'Aquitaine	500	250	302
Belgian Blue	500	129	189
Charolais	500	250	1000
Friesian	500	250	249
Hereford	500	250	1000
Holstein	500	250	1000
Limousin	500	250	1000
Parthenaise	500	73	220
Saler	500	250	1000
Shorthorn	500	250	995
Simmental	500	250	1000

#### Purebred training population

Within breed identity-by-state (IBS) clustering was performed on all purebred animals in Plink V1.9 (Purcell et al., 2007), which investigates whether animals share zero, one, or two alleles at each locus across the genome. IBS clustering was used to identify the most genomically diverse animals within each breed to represent the training population. Within each breed, 500 clusters were created, and animals that had similar genomes were grouped together. One animal was randomly chosen from each cluster to represent the purebred training population for breed assignment. The purebred training population was established to calibrate models for predicting breed composition.

#### Purebred and admixed validation populations

To validate whether breed composition could be predicted using SNP data, a population of purebred and crossbred animals which had no direct relationship (i.e., parent-offspring and vice-versa) to the purebred training population was generated. Where possible, 250 purebred-verified animals from each of the 13 breeds were included in the validation population.

In order to identify a known admixed population for validating SNP-BLUP and Admixture breed composition predictions, a supervised Admixture analysis (*K = 13*) was completed on all genotyped animals. The 500 purebred animals within each of the 13 breeds from the training population were fixed as purebred in a supervised Admixture analysis and the breed composition of all remaining admixed animals was predicted. Animals compromised of, at most 4 breeds were subsequently selected where each of the breeds represented had to belong to one of the 13 purebred populations included in the present study. In the two-way crosses, animals which had an Admixture breed composition prediction between 45%–55%:45%–55%, 20%–30%:70%–80% or 70%–80%:20%–30% were retained as a two-way validation population, consisting of 2,281 animals. Animals with an admixed breed composition comprised of ≥ 20% for each of three separate breeds and <2.5% of a fourth breed were also included in a separate three-way cross validation population, consisting of 2,059 animals. A summary of the number of animals per breed in the crossbred validation population is in Table 2.

**TABLE 2:** Number of animals in the crossbred validation population.

Crosses	Breed^a^	Number
2 Way Cross (*n* = 2,281)	AA × CH	998
	AA × HE	144
	AA × SI	311
	CH × LM	140
	HO × FR	233
3 Way Cross (*n* = 2049)	AA × HO × FR	474
	AA × BA × LM	180
	AU × BA × LM	1280
	SI × HO × FR	80
	SI × SH × CH	55

^a^ AA, Angus; AU, Aubrac; BA, Blonde d’Aquitaine; BB, Belgian Blue; CH, Charolais; FR, Friesian; HE, Hereford; HO, Holstein; LM, Limousin; SH, Shorthorn; SI, Simmental.

#### Purebred SNP selection population

An additional purebred SNP selection population was established in order to quantify the information content of individual SNPs in predicting breed composition; this was necessary to rank the SNPs for the development of low-density panels. This SNP selection population consisted of 1,000 purebred animals per breed where possible that were not included in the purebred training or validation populations. The number of animals per breed included in the SNP selection population was capped at 1,000 where possible in order to keep a relatively similar number of animal per breed. This SNP selection population consisted of 9,955 purebred animals (Table 1).

### Divergence among breeds

The pairwise $F_{st}$ statistic represents a measure of the genetic distance among breeds (Weir and Cockerham, 1984). The pairwise fixation indexes ( $F_{st}$ ) were calculated for the SNP selection population in a supervised Admixture (*K = 13*) analysis as:

$$F_{st}=\frac{s^{2}}{\bar{p}\left(1-\bar{p}\right)}$$

where $s^{2}$ is the standard deviation (SD) of the allele frequency among breeds and $\bar{p}$ is the mean allele frequency across breeds (Weir and Cockerham, 1984). A phylogenetic tree was computed using the breed pairwise $F_{st}$ scores with the APE package in R software (Paradis et al., 2004) to visualise the genetic differentiation among all 13 breeds.

### Breed composition estimated using single nucleotide polymorphisms best linear unbiased prediction

SNP-BLUP using MIX99 software (Mix99 Development Team, 2017) was used to estimate the breed composition of animals in the validation population, with the results compared to breed composition estimates from Admixture (Alexander et al., 2009). The SNP-BLUP approach followed the pipeline described by O’Brien et al. (2020) for predicting breed composition in sheep using SNP genotypes. All SNPs were fitted as random effects which were assumed to be identically and independently distributed with mean zero and common variance structure N (0,**I** $\sigma_{g}^{2}$ ):

$$y_{i}=\mu +\sum_{i=1}^{n}X_{j}g_{ij}+e_{i}$$

where the dependant variable $y_{i}$ was coded as either one if the animal was in the training population for the breed under investigation or zero if the animal was in the training population but not for the breed under investigation. The number of animals coded as purebred for each breed was equal to the number of animals coded as non-purebred for that breed. For example, 500 animals were classified as purebred Angus and coded as 1, while from the 12 remaining breeds, 500 animals were randomly selected such that each of the 12 breeds were equally represented. These 500 animals from the other 12 breeds were coded as 0, i.e., not Angus. All remaining animals were classified as missing. The intercept is denoted by µ, $X_{j}$ is the allele substitution effect of ${SNP}_{j}$ ; $g_{ij}$ is the random effect of the genotype of animal ⅈ at locus j and $e_{i}$ is the random effect of residual term for animal ⅈ , with the common variance structure N (0,**I** $\sigma_{e}^{2}$ ). The phenotypic SD for the dependent variable was estimated as $\sqrt{pq}$ , where p was the proportion of animals which were verified to be the breed under investigation (i.e., coded as 1); q was 1 minus this proportion. The genetic SD was estimated from the phenotypic SD assuming a heritability of 0.999 (O’Brien et al., 2020). The SNP effects obtained were subsequently multiplied by the allele count of each animal to generate estimates of breed proportion.

All subsequent breed predictions <0.05 were set to 0. The sum of all predicted breed compositions for each animal were rescaled as per O’Brien et al. (2020), where each animal’s breed proportion estimated for the breed under investigation was divided by the sum of that animal’s breed proportions estimated for all 13 breeds. Purebreds in the validation population were considered assigned if the prediction of breed composition was ≥ 0.90 for any single breed. The SNP-BLUP approach was run for a series of different genotype panels constructed (described later) as well as the entire dataset (i.e., 49,213 SNPs).

### Breed composition estimated using Admixture

Using the same training and validation populations and all 49,213 SNPs, a supervised analysis (*K* = 13) was conducted in Admixture (Alexander et al., 2009). In the Admixture analysis, the same purebreds that were used in the SNP-BLUP analysis were set as purebreds for that breed, and the breed composition of the animals in the validation population was estimated. All breed proportion estimates < 0.05 were fixed to 0 and the estimated breed proportions rescaled as with the SNP-BLUP method. Again, if the predicted breed proportion for any single breed in the purebred validation population was ≥ 0.90, purebreds were regarded as being assigned to that breed.

### Development of low-density genotype panels

Seven alternative low-density panels (i.e., 100, 500, 1,000, 2,000, 3,000, 5,000 and 7,500 SNPs) were generated using seven different SNP selection strategies. The SNP selection population (Table 1) was used to rank SNPs based on potential informativeness for the generation of these low-density panels. The number of SNP chosen per chromosome remained constant for each of the seven SNP selection methods evaluated and was proportional to the genome length of each chromosome (Supplementary Table S1). The seven alternative methods used to generate the panels were as follows.

#### Random selection

The number of predefined SNP required per chromosome was randomly selected until each of the respective panel densities was obtained.

#### Partitioning-around-medoids (PAM)

The partitioning-around-medoids (PAM) algorithm clusters SNPs on each chromosome together based on their proximity in genomic position, not taking LD into account. The algorithm was run for each chromosome separately with the number of clusters created per chromosome set to the number of predefined SNPs for that chromosome. The SNP located in the middle of each cluster was selected, as described by Lashmar et al. (2021) when developing low-density panels to assess imputation accuracy in cattle. The PAM algorithm was implemented in the R package *“cluster”* (V2.1.2 Maechler et al., 2021).

#### Fixation index ( $F_{st})$

The fixation index ( $F_{st}$ ) is used to evaluate the extent of genetic divergence between populations and identify genomic regions under selection pressure. The global $F_{st}$ was estimated using the method proposed by Weir and Cockerham (1984) across all 13 breeds in Plink V1.9 (Purcell et al., 2007) from the SNP selection population using all 49,213 SNPs. Three alternative strategies to picking SNPs based on the calculated $F_{st}$ statistic were investigated;

a) $F_{st}$ and block method: Each chromosome was divided into blocks of SNPs with one SNP chosen per block. The number of blocks on each chromosome was equal to the number of predefined number of SNPs for that chromosome. The SNP with the highest $F_{st}$ statistic within each block was chosen.
b) $F_{st}$ and PAM method: The SNP with the highest $F_{st}$ within each PAM cluster already generated previously per chromosome was selected.
c) Highest ranking SNPs based on $F_{st}$ statistics: SNPs in the n^th^ highest ranking for the $F_{st}$ statistic were chosen per chromosome, irrespective of location on that chromosome, where n was the number of predefined number of SNPs for that chromosome.

#### PCA

SNP weightings were calculated using the “smartpca” algorithm in Eigensoft v7.2.1 (Patterson et al., 2006) applied to the SNP selection population. The greater the difference in allele frequency between populations, the greater the SNP weighting. Three alternative methods of picking SNPs based on PCA ranking were investigated similar to the $F_{st}$ approach already described;

a) PCA ranking and block method: The SNP with the highest SNP weighting within each block was chosen.
b) PCA ranking and PAM method: The SNP with the highest SNP weighting within each PAM cluster was selected.
c) Highest ranking SNPs based on PCA: SNPs in the n^th^ highest ranking based on PCA SNP weightings were chosen per chromosome, irrespective of location on the chromosome, where n was the number of predefined number of SNPs for that chromosome.

#### SNP-BLUP variance

SNP-BLUP was used to estimate the SNP effects within the SNP selection population of each breed individually using all 49,213 SNPs. From this, the standard deviation (SD) of the BLUP model solutions per SNP were estimated within the SNP selection population of all 13 breeds and SNPs were ranked based on the SD of the SNP effect across all 13 breeds; SNPs with a larger standard deviation were given a higher ranking. Three alternative methods of picking SNPs based on using the SNP-BLUP SD were investigated.

a) SNP-BLUP variance and block method: The SNP with the largest standard deviation of SNP effects within each block was chosen.
b) SNP-BLUP variance and PAM method: The SNP with the largest standard deviation of SNP effects within each PAM cluster was selected.
d) Highest ranking SNPs based on SNP-BLUP variance: SNPs in the n^th^ highest ranking based on the standard deviation of SNP effects were chosen per chromosome, irrespective of location on the chromosome, where n was the number of predefined number of SNPs for that chromosome.

#### Random Forest

Random Forest is a machine-learning method (Breiman, 2001) that employs decision trees, which are a set of rules for splitting data in a way that minimises variation. The Random Forest analysis was conducted in the R package random forest (Liaw and Wiener, 2001) using the genotypes of the SNP selection population to predict the dependant variable, which was breed, and was numbered 1 to 13. The built-in variable importance measures (VIM) ranked the SNPs according to their relevance for predicting breed. The highest ranking SNPs of a predefined number per chromosome were retained.

#### PLSDA

Partial least square discriminant analysis (PLSDA) is another machine learning method based on the PLS approach (Barker and Rayens, 2003). In the present study, a PLSDA regression model was constructed using the purebred SNP selection population and their corresponding genotypes in the R package Caret (Kuhn, 2020) for discriminative SNP selection. The dependant variable was breed, and was coded numerically as +1 or −1. If an animal was a member of the breed class under analysis, that animal was coded as +1, which is referred to as the ‘in-group’ and it it was a different breed group it was coded as −1, representing the ‘out-group’ (Brereton and Lloyd, 2014)**.** The regression model was run 13 times, once for each breed. Each SNP received a weighting, and SNPs which were the most informative for distinguishing between breed classes ranked highest. The highest ranking SNPs of a predefined number per chromosome were retained.

### Evaluating the difference in breed composition predictions using the low-density panels

Breed composition predictions from SNP-BLUP using all 49,213 SNPs were considered the gold standard and used for comparing the prediction performance from each of the low-density panels. Animals in the purebred validation population were considered to be accurately assigned when their estimated breed proportion of a specific breed was predicted to be ≥0.90. The difference in the main breed proportion estimates for crossbred animals predicted using all the low-density panels and the gold standard 49,213 SNPs were compared. In addition, the three SNP selection methods with the smallest mean difference in breed composition predictions from the gold standard, were also used for breed composition prediction using Admixture (Alexander et al., 2009). The Admixture breed predictions using the low-density panels where then compared to those from the gold standard SNP-BLUP.

## Results

### Population structure

**FIGURE 1:**
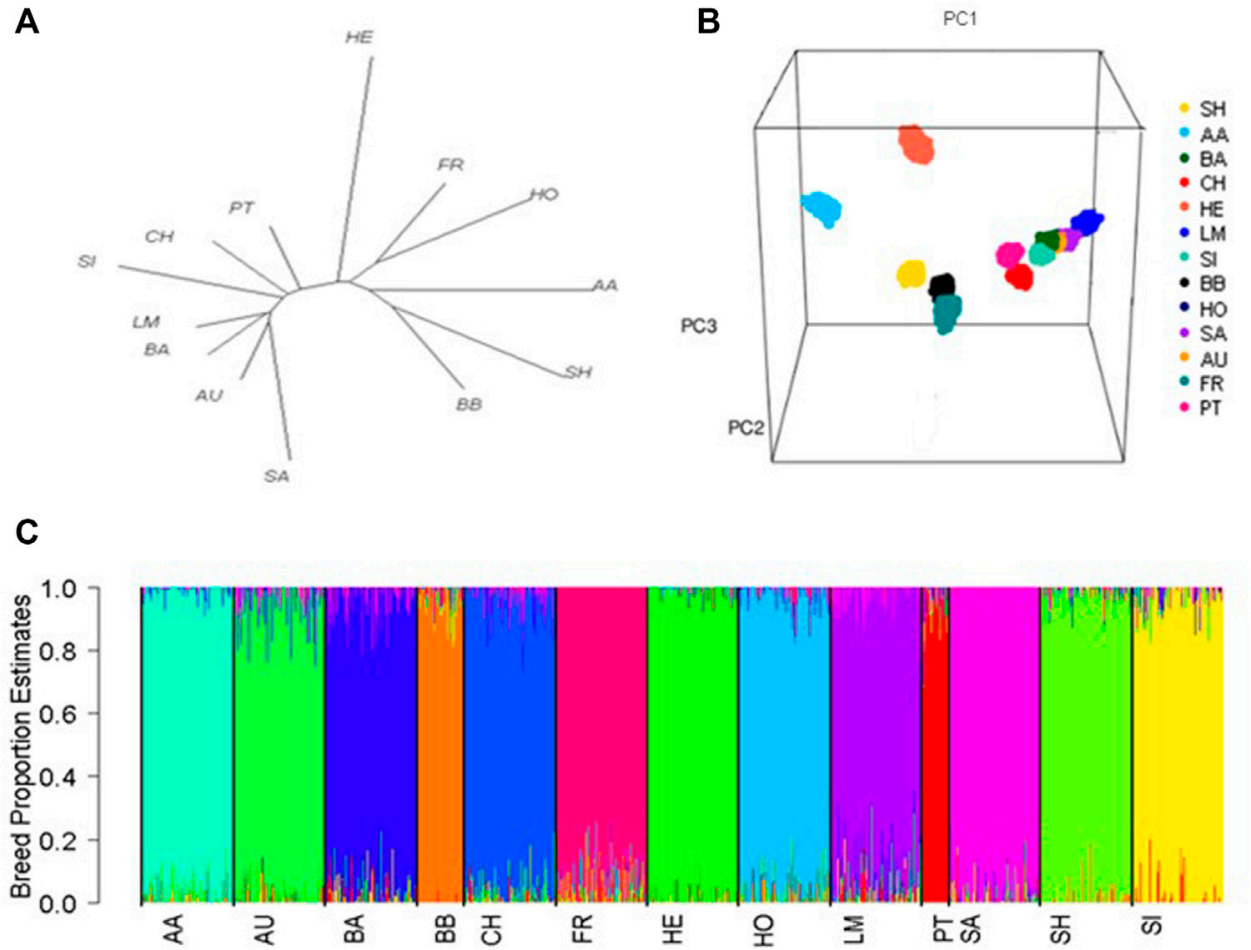
**(A)** Phylogenetic tree showing the genetic distance between breeds based on pairwise fixation index ( $F_{st}$ ) estimates, **(B)** Population distribution of purebred animals across the first three principal components (PC1, PC2, PC3), **(C)** Admixture-estimated breed proportions for each purebred animal. Each animal is represented by a thin vertical line whose length represents its breed proportion, and each colour represents an inferred population. Breeds included Angus (AA), Aubrac (AU) Blonde d'Aquitaine (BA), Belgium Blue (BB), Charolais (CH), Friesian (FR), Hereford (HE), Holstein (HO), Limousin (LM), Parthenaise (PT), Saler (SA), Shorthorn (SH), and Simmental (SI).

The greatest genetic differentiation was observed between the Salers and both the Simmental and Shorthorns ( $F_{st}$ = 0.146) while the least genetic divergence existed between the Charolais and Blonde d'Aquitaine ( $F_{st}=$ 0.039) (Supplementary Table S2). The strong genetic relationship between Aubrac, Blonde d'Aquitaine, and Limousins was also demonstrated by their shared branch in the phylogenetic tree, with Simmentals situated on the neighbouring branch (Figure 1). The PCA succesfully seperated out 13 breed clusters based on genomic data with the first, second and third principal components accounting for 22.1%, 15.7% and 13.6% of the variance, respectively. Within the PCA plot, Herefords were distinctly separated from other breeds, confirming their high $F_{st}$ value relative to other breeds (Kuehn et al., 2011; Kelleher et al., 2017). The close genetic relationship between Simmental, Blonde d’Aquitaine, Aubrac and Limousin was again evident through the close proximity of their respective breed clusters (Supplementary Figure S1).

### Breed composition prediction

**FIGURE 2:**
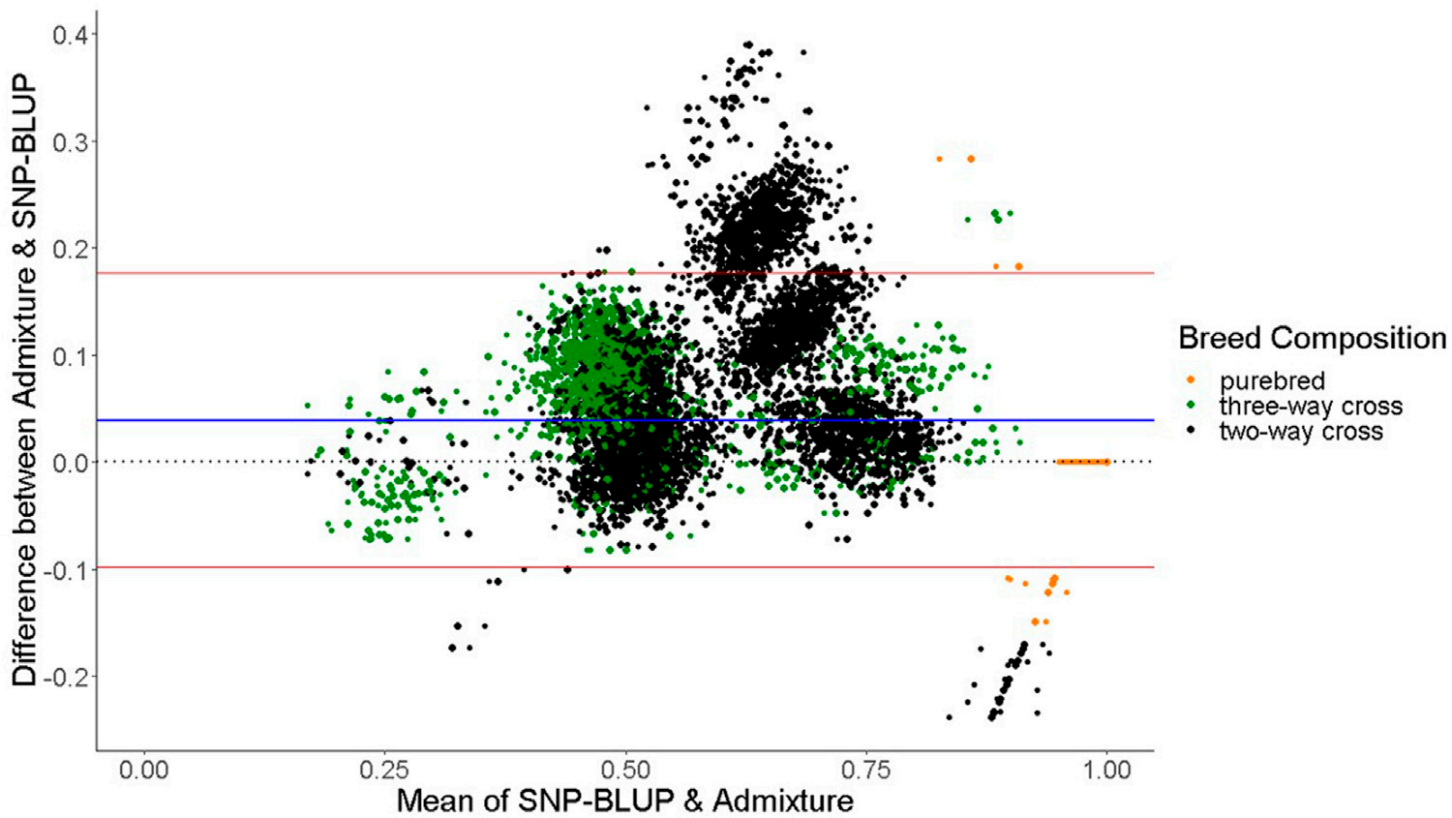
Bland-Altman plot displaying the differences (y-axis) against the mean of the values (x-axis) of SNP-BLUP and Admixture breed proportions for two-way cross, three-way cross, and purebred animals. The horizontal red lines represent the mean ± 2 standard deviations and the horizontal blue line represents the mean difference between Admixture and SNP-BLUP prediction of breed composition.

The mean difference in predicted breed composition between SNP-BLUP and Admixture using all 49,213 SNPs was 0.04 across both purebred and crossbreds, which was not different (*p* > 0.05) from zero, suggesting that there is no systematic difference between the methods that would lead to over or underestimation of breed composition. Both SNP-BLUP and Admixture accurately assigned ≥ 98% of the purebred validation population to the correct breed. When comparing the prediction of breed composition of each breed individually, the largest difference observed in predictions between SNP-BLUP and Admixture for the purebreds was for the Belgian Blue (0.004) while no mean difference was detected for Angus, Aubrac, Charolais, Friesian, Hereford, Holstein, Limousin, Salers, Shorthorn and Simmental (Table 3). For both purebred and crossbreds in the validation population, the variability in predicted breed composition from SNP-BLUP and Admixture is shown in a Bland-Altman plot (Figure 2). In comparison to purebred predictions, a larger absolute mean difference in predicted breed composition was observed in the crossbred validation population, with an average absolute mean difference of 0.08 and 0.05 for the two and three-way cross validation animals, respectively (Table 4). Ninety percent of the SNP-BLUP and Admixture breed composition predictions differed by less than 0.14. Of all the crossbred animals, the biggest discrepancy between SNP-BLUP and Admixture breed composition predictions was for Holstein-Friesian two-way cross animals.

**TABLE 3:** Mean absolute difference and standard deviation of the difference of the absolute values between the SNP-BLUP and Admixture breed predictions for the purebred validation population in each breed.

Breed	Mean difference	Standard deviation
Angus	0.000	0.000
Aubrac	0.000	0.000
Blonde d'Aquitaine	0.003	0.023
Belgian Blue	0.004	0.037
Charolais	0.000	0.000
Friesian	0.000	0.007
Hereford	0.000	0.000
Holstein	0.000	0.000
Limousin	0.000	0.000
Parthenaise	0.003	0.021
Saler	0.000	0.000
Shorthorn	0.000	0.007
Simmental	0.000	0.000

**TABLE 4:** Mean absolute difference and standard deviation of the difference of the absolute values between the SNP-BLUP and Admixture breed predictions for the crossbred validation population.

Breed	Mean difference	Standard deviation
Angus	0.062^a^	0.041
Belgian Blue	0.057	0.067
Charolais	0.035^a^	0.031
Friesian	0.158^a^	0.111
Hereford	0.046^a^	0.032
Holstein	0.155^a^	0.068
Limousin	0.031^a^	0.042
Shorthorn	0.024	0.023
Simmental	0.051^a^	0.039

^a^ Difference is significantly different from zero.

### Low-density panel predictions of breed composition with SNP-BLUP

#### Purebred predictions

**FIGURE 3:**
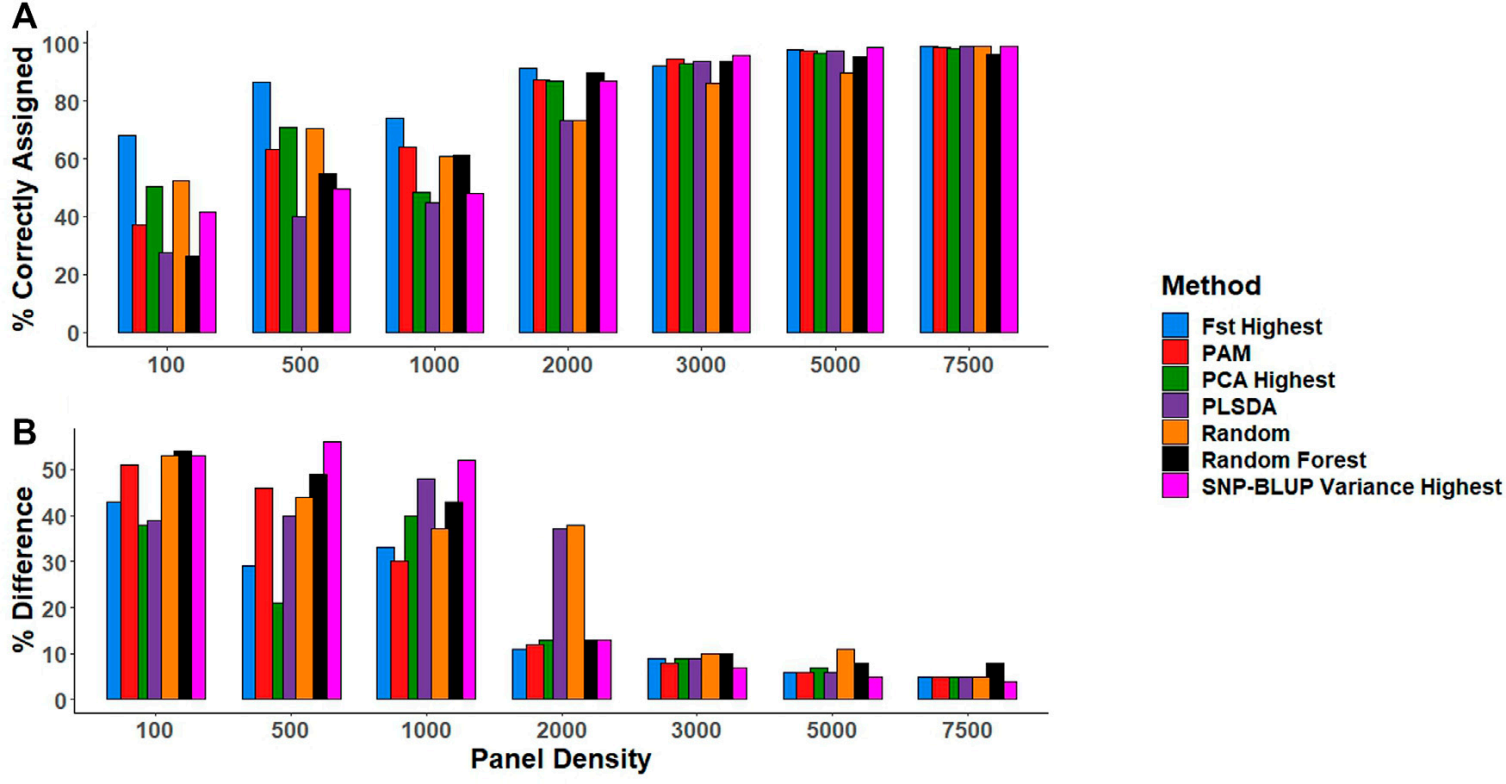
**(A)** The percentage of animals in the purebred validation population correctly assigned to the respective breed (i.e., predicted to have a breed proportion >0.9 for their respective breed). **(B)** The percentage difference between the gold standard (estimates using all 49,213 SNPs) and low-density breed proportion estimates for crossbreds. SNP selection methods for the creation of low-density panels included pairwise fixation index highest (Fst highest), partitioning-around-medoids (PAM), principal component analysis highest (PCA highest), partial least square discriminant analysis (PLSDA), random SNP selection (Random), Random Forest, and SNP-BLUP variance highest.

In general, the number of correctly assigned purebreds increased with increasing panel density across all SNP selection strategies (Figure 3). All SNP selection strategies correctly assigned >85% of the purebred validation population when the SNP density was ≥2,000 SNPs, with the exception of the PLSDA and Random selection method, which both required a minimum of 3,000 SNPs to correctly assign >85% of the purebred validation population to their respective breeds (Figure 3).

#### Crossbred predictions

Similarly, as panel density increased the mean difference between the gold standard breed composition estimates and breed prediction estimates using the low-density panels reduced for crossbreds (Figure 3). The estimation of crossbred breed composition was more challenging than that of purebreds, requiring a minimum of 3,000 SNPs for accurate crossbred breed composition predictions across the different SNP selection strategies, regardless of whether they were two or three-way crosses (Figure 3). When panel density was ≥ 3,000 SNPs, breed composition estimates deviated from the gold standard by an average of 0.055 and 0.079 for two and three-way crosses, respectively.

### Comparison of SNP selection strategies

There was little overlap in the actual SNPs selected by each SNP selection strategy (Supplementary Figure S2). There was a minimal difference in performance between the SNPs selected using the various SNP selection methods for predicting breed composition at panel densities ≥ 3,000 SNPs. At panel densities <3,000 SNPs, SNPs selected using the $F_{st}$ method most accurately predicted breed composition, followed by the PCA selection strategy (Figure 3). Interestingly, when the genomic position of the SNP was considered in the $F_{st}$ and PCA SNP selection methods (i.e., the block and PAM method), breed composition estimates were considerably less accurate than the $F_{st}$ and PCA SNP selection method where position was not taken into account (Figure 3). When comparing machine learning methods across densities, in general, SNPs selected using Random Forest were better at predicting the breed composition of both purebreds and crossbreds than SNPs selected using PLSDA (Figure 3).

### Comparison with Admixture predictions

**FIGURE 4:**
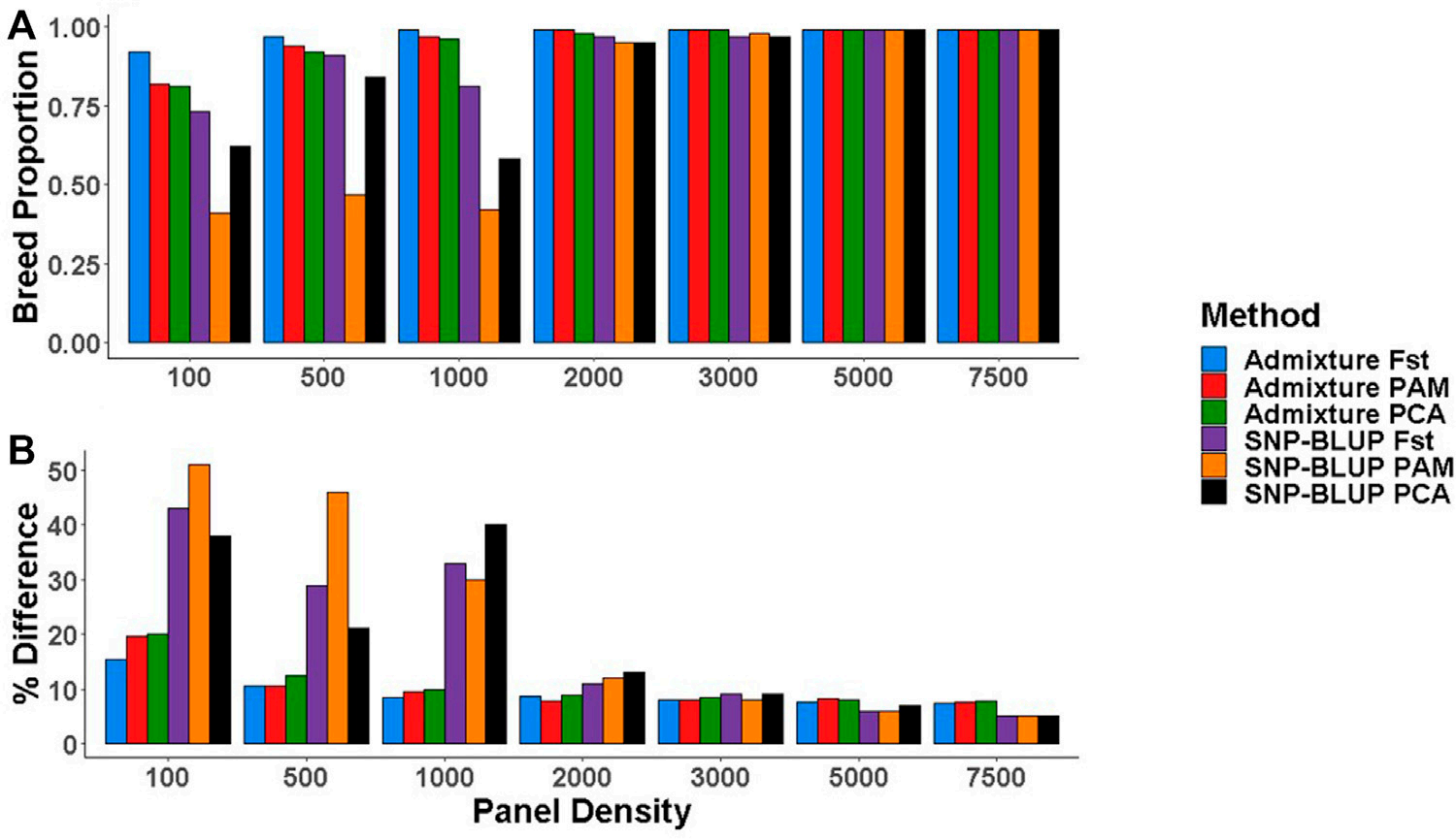
**(A)** Purebred breed proportion estimates from Admixture and SNP-BLUP using low-density panels, **(B)** The percentage difference between the gold standard (estimates using all 49,213 SNPs) and low-density breed proportion estimates from Admixture and SNP-BLUP. SNP selection methods for the creation of low-density panels included pairwise fixation index highest (Fst), partitioning-around-medoids (PAM), and principal component analysis highest (PCA).

When 49,213 SNPs were used, there was no systematic difference between the breed composition predictions by SNP-BLUP *versus* Admixture. SNPs selected using the three most accurate SNP selection methods (i.e., $F_{st}$ highest, PCA highest, and PAM) for the creation of low-density panels were also used for predicting breed composition in Admixture (Alexander et al., 2009). Admixture proved to be more accurate at predicting breed composition than SNP-BLUP when panel density was <2,000 SNPs. Breed composition estimated from Admixture had an absolute mean difference of 0.091 from the gold standard SNP-BLUP breed composition predictions, whereas estimates from SNP-BLUP had an absolute mean difference of 0.315 from the gold standard estimates. Admixture required fewer SNPs than SNP-BLUP to accurately predict breed composition, with 500 and 1,000 SNPs sufficing to accurately predict the breed composition of purebred and crossbred cattle, respectively, whereas SNP-BLUP required 2,000 and 3,000 SNPs (Figure 4). Across both SNP-BLUP and Admixture, SNPs selected using the $F_{st}$ highest SNP selection method generally preformed best at predicting breed composition.

## Discussion

The objective of the present study was to compare SNP-BLUP and Admixture as methods to predict the breed composition of purebred and crossbred cattle; of particular interest also was to investigate if the accuracy of predicting breed composition was eroded as SNP density reduced, and also if the approach to select these SNP impacted the conclusion. Marginal differences existed between both breed prediction methods once genotypes from >2,000 informative SNPs were available on all animals. Moreover, once animals were genotyped for >3,000 SNPs (which is generally the norm in cattle), how these SNPs were selected did not impact greatly the predictions.

### SNP-BLUP vs. Admixture

The only discrepancies observed between SNP-BLUP and Admixture breed composition predictions for purebreds using the full SNP dataset was for Blonde d'Aquitaine, Belgian Blue, and Parthenaise. Some animals in these breeds were misassigned to another closely related breed; this phenomenon may be due to the relatively smaller sample sizes of these breeds in the validation population (i.e., sampling variability) as well as their close genetic resemblance to other breeds. The largest discrepancy in prediction of breed composition detected between Admixture and SNP-BLUP predictions for crossbred animals was for the two-way Holstein-Friesian crosses (Figure 2); because these two breeds are genetically similar, differentiating which portion of the genome is attributed to Holstein and which attributed to Friesian was challenging. Because the Holstein and Friesian compositions in a three-way cross animal represented a smaller proportion of the animal’s overall breed composition, lesser differences between Admixture and SNP-BLUP predictions were evident in three-way crosses with a Holstein-Friesian component than in two-way crosses comprised of exclusively Holstein and Friesian. It should also be noted that only animals with a breed composition made up of at most four breeds were included in the crossbred validation population. The rationale behind this was that animals with more than four breeds in their genome might have experienced degraded breed haplotypes inherited from their ancestors over time, which would likely make predicting their breed composition particularly challenging.

In order to assess how mislabeled individuals in the training population affect breed composition predictions using SNP-BLUP and Admixture, 250 purebred Angus animals were substituted with 250 two-way cross Angus animals in the training population; they were all labelled as purebred Angus. All 250 purebred Angus animals in the validation population were predicted by Admixture to be ≥0.90 Angus. In contrast, when predictions were based on SNP-BLUP, only six of the 250 purebred Angus animals in the validation population were predicted to be ≥0.60 Angus. Therefore, SNP-BLUP appears to be more sensitive to mislabeled individuals in the training population, while Admixture was able to accurately estimate breed composition even with 250 (i.e., half the population) mislabeled individuals present.

### Low-density panels

SNP-BLUP required a minimum of 2,000 and 3,000 SNPs to accurately predict purebred and crossbred breed composition, respectively; the respective values for Admixture was 500 and 1,000 SNPs, corroborating results of Bjørnstad and Røed (2002) who concluded that assigning crossbred (horses) to the correct breed using the frequency method outlined by Paetkau et al. (1995) is more challenging than that of purebreds. It should be noted that in the present study, a separate population was used to select SNPs for the creation of the low density panels so as to minimise SNP selection bias. Strucken et al. (2017) emphasised the importance of utilising a separate population for the selection of SNPs for predicting breed composition, reporting that when the prediction equations were not generated from a population independent of the test dataset, it resulted in a substantial increase in ascertainment bias.

Many factors impact the number of SNPs required for the accurate prediction of breed composition. These factors include, but are not limited to, the breeds included in the study (Lewis et al., 2011; Hulsegge et al., 2013), given that breeds which are closely related are likely to have similar allele frequencies and therefore be more difficult to differentiate than breeds which are not genetically similar (Wilkinson et al., 2011; Kavakiotis et al., 2015). In addition, the effective population size of the population also contributes to the number of SNPs needed to accurately determine breed composition as populations with larger effective population sizes are genetically more diverse. The effective population of the breeds in the present study were previously estimated by McParland et al. (2007) to range between 64 and 127 per breed. Similar estimates in cattle have been reported elsewhere (Stachowicz et al., 2011; Rodríguez-Ramilo et al., 2015; Doekes et al., 2018) and are a reflection of the intense selection and genetic drift breeds have been subjected to. SNP selection methods therefore that choose the most informative SNPs for breed prediction require fewer SNPs for accurate breed composition predictions than using less or non-informative SNPs (Ding et al., 2011; Chhotaray et al., 2019) such as using the random SNP selection method, as demonstrated in the present study. The number of SNPs necessary for accurate breed composition predictions also depends on whether Admixture or a regression model such as SNP-BLUP is used for predictions, with Admixture requiring fewer SNPs than regression models (Strucken et al., 2017; He et al., 2018; Reverter et al., 2020).

### SNP selection methods for low-density panels

The success of the F_st_ SNP selection method for identifying informative SNPs which can be used for the prediction of breed composition in cattle has been extensively reported previously (Lewis et al., 2011; Wilkinson et al., 2011; Hulsegge et al., 2013; Bertolini et al., 2015), as has the PCA SNP selection method (Paschou et al., 2007; Lewis et al., 2011; Bertolini et al., 2015; Chhotaray et al., 2019). Unlike some previous studies (Ding et al., 2011; Hulsegge et al., 2013; Strucken et al., 2017), a linkage disequilibrium (LD) threshold or minimum distance between selected SNPs was not implemented when creating low-density panels in this study as no prior assumptions were made about which SNPs may or may not be informative for breed prediction. As a result, the F_st_ and PCA highest methods both chose SNPs located in close proximity and consequently in strong LD on each autosome, particularly when panel density was ≤ 1,000 SNPs (Supplementary Figure S3). This was not surprising, as previous literature also reported the PCA (Paschou et al., 2007; Lewis et al., 2011; Bertolini et al., 2015) and F_st_ method (Wilkinson et al., 2012) of ranking SNPs to be susceptible to choosing SNPs in strong LD with each other. Despite the strong LD observed between the SNPs chosen by the PCA and F_st_ highest methods, these SNP selection strategies performed better at predicting breed composition than SNPs selected using the other SNP selection methods evaluated, all of which had weaker LD among SNPs. This suggests that informative SNPs for the prediction of breeds may be in LD and cluster together in close proximity along the genome, and the benefit of increasing SNP panel density was less with the PCA and F_st_ highest methods in comparison to the other methods that had weaker LD among SNPs. Wilkinson et al. (2012) noticed a similar trend, and deduced that a strong level of LD when designing low-density panels could be a signature reflecting positive selection as result of modern breeding programmes, and that these SNPs may show strong breed differentiation due to positive selection for breed-specific characteristics. Consequently, despite recommendations to remove SNPs in LD prior to Admixture or PCA analysis (Novembre et al., 2008; Alexander et al., 2009; Mattucci et al., 2019; Dutheil, 2020), SNPs in LD could potentially be highly informative for breed composition prediction, particularly when SNP density was low (Wilkinson et al., 2012).

Although machine learning algorithms have been widely applied to cattle breeding for the prediction of a wide variety of traits such as lameness (Warner et al., 2020), longevity (Van Der Heide et al., 2019) and milk composition (Gianola et al., 2011; Frizzarin et al., 2021), these algorithms have not been utilized extensively in predicting breed composition in cattle. Prior research has reported that machine learning does not predict certain traits in cattle and sheep as effectively as other traditional methods such as regression models (Cortez et al., 2006; Van Hertem et al., 2014; Hempstalk et al., 2015), corroborating the findings of the present study. Previous literature has reported that the majority of PLSDA models suffer from overfitting (Westerhuis et al., 2008) and inconsistent performance (Szymańska et al., 2012). While Bertolini et al. (2015) successfully used Random Forest in conjunction with PCA for SNP selection and breed assignment in cattle, the accuracy of this method was based on the percentage of animals assigned to the correct breed, whereas accuracy in the present study was based on the more difficult task of assigning breed proportions and predicting the overall breed composition of individual cattle. Another key difference between the present study and that of Bertolini et al. (2015) is that the present study only used Random Forest for SNP selection and used SNP-BLUP and Admixture for breed proportion predictions, whereas Bertolini et al. (2015) used Random Forest to select informative SNPs, before fitting a new Random Forest algorithm to determine breed assignment.

The little overlap in SNPs selected across SNP selection approaches is likely due to the difference between the SNP selection methods used. Out of all SNP selection methods investigated, Random Forest was the only one that considered possible correlations among SNPs. F_st_-based selection focused on the standardized variance in allele frequency among populations, while PCA-based selection focused on patterns in the data, identifying SNPs that had high loadings on the first three principal components, which captured the most significant patterns in the data. On the other hand, the PAM method only considered the genomic position of the SNP. Schiavo et al. (2020) also reported little overlap in SNPs selected when comparing SNPs selected using the F_st_, PCA and Random Forest methods.

### Training population

It should be noted that ensuring purebred animals are recorded correctly and a careful selection of the most genetically diverse animals within breed to be included in the training population is crucial. As suggested by others (Bjørnstad and Røed, 2002; Dalvit et al., 2008), when predicting breeds, some animals may never be correctly assigned regardless of the number of SNPs used because the breeds are too genetically similar or because the individuals are genetically atypical for their breeds. To avoid the latter from happening, and ensure maximum prediction accuracy, the training population in the present study consisted of very large numbers of animals in comparison to previous similar studies (Lewis et al., 2011; Wilkinson et al., 2011), increasing the within-breed variability. Bertolini et al. (2015) advocated that the more animals included in the training population the greater the within breed variability captured, possibly resulting in an enhanced performance for breed prediction. While previous studies randomly selected purebreds to represent their training population, Hulsegge et al. (2013) noted that for the optimum prediction of breed composition, when selecting the training population, it is crucial to choose the most genetically diverse animals within each breed. Bearing this in mind, a novel approach was implemented in the present study, utilising IBS clustering to aid with selecting the most genetically dissimilar animals to represent the training population for each breed. IBS clustering compares two individuals which share 0, 1 or 2 alleles at a given locus throughout the genome (Stevens et al., 2011), grouping genetically similar animals together. Randomly selecting one animal from each genetically different IBS cluster to represent the training population ensured that the training population captured the majority of the variation of genotypes in each breed.

### Applications

The present study demonstrated that genomic information can be utilised in generating accurate predictions of breed composition which could potentially be useful for increasing the accuracy of genetic evaluations by being better able to fit breed covariates in an admixed population. Sevillano et al. (2017) confirmed the superior performance of genomic evaluation models that account for breed-specific SNP effects in admixed populations compared to those assuming uniform SNP effects across breeds. This suggests that the accurate determination of breed composition can enhance genomic predictions. Furthermore, accurate breed composition information could also potentially be utilised in quality control of genotypes entering the database and to further augment various breeding strategies for improvement of cattle breeds.

## Conclusion

There was a strong similarity in predicted breed composition per animal between the SNP-BLUP and Admixture approaches investigated when panel density was ≥3,000 SNPs. This suggests that the prediction of breed composition could be readily integrated into the SNP-BLUP pipelines used for genomic evaluations thus replacing the use of a stand-alone software. Despite approximately 50,000 SNPs existing on most routinely-used genotyping panels, only small subsets of highly informative SNPs are required to accurately predict breed composition. This study provides a blueprint for the utilisation of the readily available next-generation sequencing technologies in the prediction of breed composition, by offering possible methods for how to identify the most informative SNPs and the optimum panel density. In general, SNPs selected using the F_st_ highest approach performed the best in terms of predicting purebred and crossbred breed composition, but only a marginal difference was observed between the performance of SNPs selected across all SNP selection methods when ≥ 3,000 SNPs were included in the analysis. This indicates that at this SNP density, all SNP selection methods could be a powerful computational time saving tool for the accurate prediction of purebred and crossbred breed composition.

## Data Availability

The data analyzed in this study is subject to the following licenses/restrictions: The genotypes used are owned by the Irish Breeding Cattle Federation (ICBF). Requests to access these datasets should be directed to https://www.icbf.com/.
