# Supplementary material for: Evaluating the use of statistical and machine learning methods for estimating breed composition of purebred and crossbred animals in thirteen cattle breeds using genomic information
Source: Front Genet. 2023 May 15;14:1120312. doi: 10.3389/fgene.2023.1120312 (PMC10237237; doi:10.3389/fgene.2023.1120312)
Supplement: Supplementary file 1 [file DataSheet1.docx]

## Supplementary Figures and Tables

**Supplementary Table 1.** Number of SNP chosen per chromosome for each of the seven low-density genotype panels.

|  | SNP density | | | | | |  |
| --- | --- | --- | --- | --- | --- | --- | --- |
| Chromosome | 500 | 1000 | 2000 | 3000 | 4000 | 5000 | 7500 |
| 1 | 31 | 63 | 125 | 188 | 251 | 314 | 470 |
| 2 | 26 | 52 | 104 | 156 | 209 | 261 | 391 |
| 3 | 25 | 49 | 99 | 148 | 198 | 247 | 370 |
| 4 | 23 | 47 | 94 | 141 | 188 | 235 | 352 |
| 5 | 22 | 44 | 88 | 132 | 177 | 221 | 331 |
| 6 | 24 | 48 | 96 | 145 | 193 | 241 | 362 |
| 7 | 21 | 43 | 85 | 128 | 170 | 213 | 319 |
| 8 | 22 | 44 | 88 | 133 | 176 | 221 | 332 |
| 9 | 20 | 40 | 81 | 121 | 162 | 202 | 303 |
| 10 | 20 | 39 | 78 | 118 | 157 | 196 | 293 |
| 11 | 20 | 41 | 82 | 123 | 163 | 204 | 307 |
| 12 | 15 | 30 | 59 | 89 | 119 | 148 | 222 |
| 13 | 17 | 35 | 69 | 104 | 138 | 173 | 259 |
| 14 | 16 | 32 | 64 | 96 | 128 | 160 | 240 |
| 15 | 17 | 34 | 68 | 101 | 135 | 169 | 254 |
| 16 | 17 | 33 | 66 | 99 | 132 | 165 | 248 |
| 17 | 15 | 30 | 61 | 91 | 122 | 152 | 228 |
| 18 | 13 | 27 | 54 | 81 | 108 | 134 | 202 |
| 19 | 19 | 37 | 75 | 112 | 149 | 187 | 280 |
| 20 | 18 | 36 | 72 | 107 | 143 | 179 | 269 |
| 21 | 15 | 29 | 57 | 87 | 116 | 145 | 218 |
| 22 | 12 | 24 | 48 | 72 | 95 | 119 | 179 |
| 23 | 12 | 24 | 47 | 71 | 94 | 118 | 176 |
| 24 | 11 | 22 | 45 | 67 | 89 | 112 | 167 |
| 25 | 10 | 19 | 39 | 58 | 78 | 97 | 146 |
| 26 | 10 | 20 | 40 | 60 | 80 | 100 | 150 |
| 27 | 10 | 19 | 39 | 58 | 78 | 97 | 146 |
| 28 | 9 | 18 | 36 | 53 | 71 | 89 | 134 |
| 29 | 10 | 21 | 41 | 61 | 81 | 101 | 152 |

**Supplementary Table 2.** Pairwise fixation index ($F_{st}$) values amoung breeds^1^.

|  | AA | AU | BA | BB | CH | FR | HE | HO | LM | PT | SA | SH |
| --- | --- | --- | --- | --- | --- | --- | --- | --- | --- | --- | --- | --- |
| AA |  |  |  |  |  |  |  |  |  |  |  |  |
| AU | 0.116 |  |  |  |  |  |  |  |  |  |  |  |
| BA | 0.112 | 0.043 |  |  |  |  |  |  |  |  |  |  |
| BB | 0.107 | 0.118 | 0.094 |  |  |  |  |  |  |  |  |  |
| CH | 0.1 | 0.135 | 0.054 | 0.079 |  |  |  |  |  |  |  |  |
| FR | 0.108 | 0.092 | 0.086 | 0.089 | 0.079 |  |  |  |  |  |  |  |
| HE | 0.139 | 0.148 | 0.118 | 0.128 | 0.114 | 0.122 |  |  |  |  |  |  |
| HO | 0.12 | 0.085 | 0.097 | 0.092 | 0.089 | 0.072 | 0.135 |  |  |  |  |  |
| LM | 0.11 | 0.041 | 0.038 | 0.193 | 0.08 | 0.085 | 0.118 | 0.096 |  |  |  |  |
| PT | 0.098 | 0.081 | 0.05 | 0.08 | 0.054 | 0.074 | 0.106 | 0.107 | 0.095 |  |  |  |
| SA | 0.141 | 0.065 | 0.069 | 0.123 | 0.054 | 0.117 | 0.148 | 0.127 | 0.067 | 0.08 |  |  |
| SH | 0.107 | 0.121 | 0.115 | 0.083 | 0.129 | 0.105 | 0.144 | 0.117 | 0.114 | 0.103 | 0.146 |  |
| SI | 0.124 | 0.071 | 0.069 | 0.106 | 0.07 | 0.1 | 0.133 | 0.103 | 0.068 | 0.11 | 0.095 | 0.129 |
| ^1^Angus (AA), Aubrac (AU), Blonde d'Aquitaine (BA), Belgian Blue (BB), Charolais (CH), Friesian (FR), Hereford (HE), Holstein (HO), Limousin (LM), Parthenaise (PT), Saler (SA), Shorthorn (SH), and Simmental (SI). | | | | | | | | | | | | |


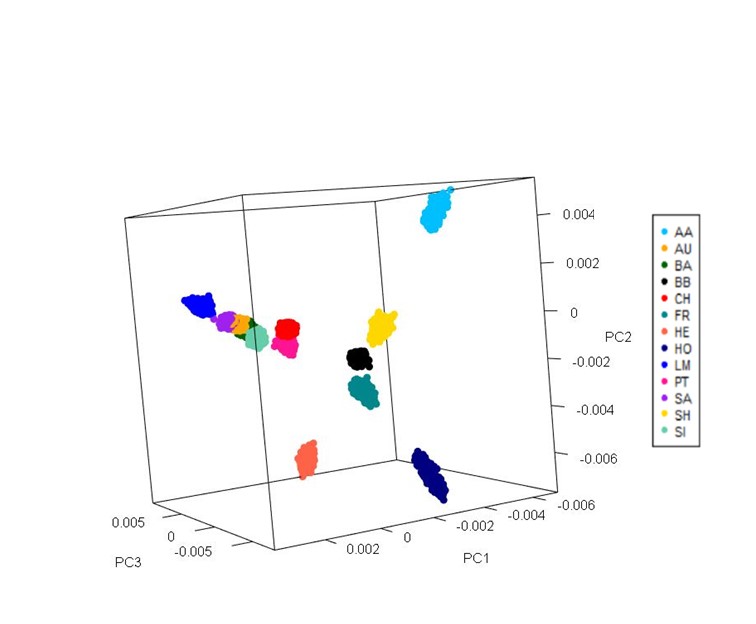


**Supplementary Figure 1.** Population distribution of all 44,802 purebred animals across the first three principal components (PC1, PC2, PC3). Breeds included Angus (AA), Aubrac (AU), Blonde d'Aquitaine (BA), Belgian Blue (BB), Charolais (CH), Friesian (FR), Hereford (HE), Holstein (HO), Limousin (LM), Parthenaise (PT), Saler (SA), Shorthorn (SH), and Simmental (SI).


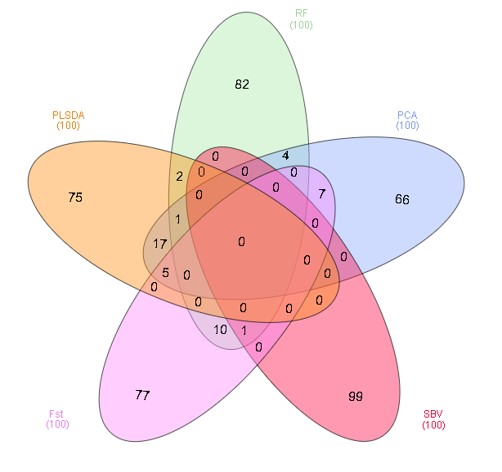
**Supplementary Figure 2**. Venn diagram of the number of SNPs selected in common between the principal component analysis highest (PCA), pairwise fixation index highest ($F_{st}$), SNP-BLUP variance highest (SBV), Random Forest, and partial least square discriminant analysis (PLSDA) SNP selection methods at 100 SNP panel density.


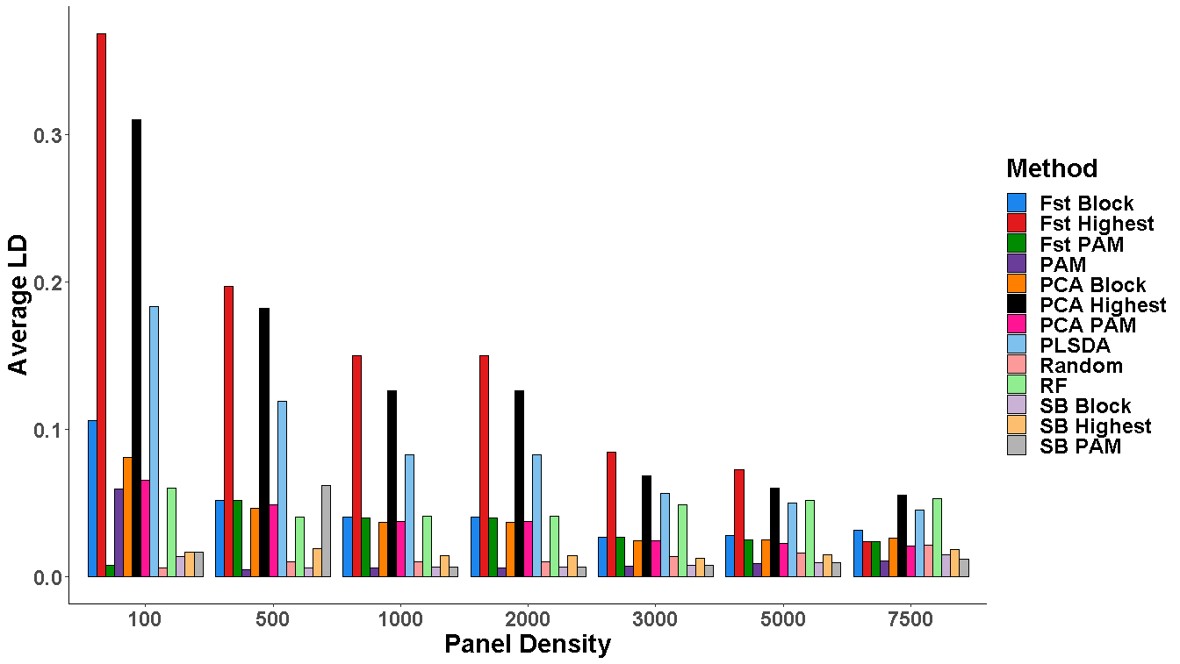


**Supplementary Figure 3.** Average linkage disequilibrium ($r^{2}$) between adjacent SNPs selected using SNP selection methods evaluated across all low-density panels. SNP selection methods for the creation of low-density panels included pairwise fixation index (Fst) block (Fst Block), Fst Highest, Fst partitioning-around-medoids (Fst PAM), PAM, principal component analysis (PCA) Block (PCA Block), PCA Highest, PCA PAM, partial least square discriminant analysis (PLSDA), random SNP selection (Random), Random Forest (RF), SNP-BLUP variance block (SB Block), SNP-BLUP variance Highest (SB Highest), SNP-BLUP variance, and PAM (SB PAM).
